# Propranolol blocks osteosarcoma cell cycle progression, inhibits angiogenesis and slows xenograft growth in combination with cisplatin-based chemotherapy

**DOI:** 10.1038/s41598-022-18324-3

**Published:** 2022-09-08

**Authors:** Luisina M. Solernó, Natasha T. Sobol, María F. Gottardo, Carla S. Capobianco, Maximiliano R. Ferrero, Liliana Vásquez, Daniel F. Alonso, Juan Garona

**Affiliations:** 1 Center for Molecular and Translational Oncology (COMTra), Science and Technology Department, National University of Quilmes, Buenos Aires, Argentina; 2 Center for Translational Medicine, El Cruce “Néstor Kirchner” Hospital, Buenos Aires, Argentina; 3 Precision Medicine Research Center, School of Medicine, University of San Martín de Porres, Lima, Perú; 4 National Council of Scientific and Technical Research (CONICET), Buenos Aires, Argentina; 5 Max Planck Institute for Heart and Lung Research, Bad Nauheim, Germany; 6 Biomedicine Research Institute of Buenos Aires (IBioBA), Buenos Aires, Argentina

**Keywords:** Cancer, Drug discovery, Oncology

## Abstract

Osteosarcoma is still associated with limited response to standard-of-care therapy and alarmingly elevated mortality rates, especially in low- and middle-income countries. Despite multiple efforts to repurpose β-blocker propranolol in oncology, its potential application in osteosarcoma management remains largely unexplored. Considering the unsatisfied clinical needs of this aggressive disease, we evaluated the antitumoral activity of propranolol using different in vitro and in vivo osteosarcoma preclinical models, alone or in addition to chemotherapy. Propranolol significantly impaired cellular growth in β2-adrenergic receptor-expressing MG-63 and U-2OS cells, and was capable of blocking growth-stimulating effects triggered by catecholamines. siRNA-mediated ADRB2 knockdown in MG-63 cells was associated with decreased cell survival and a significant attenuation of PPN anti-osteosarcoma activity. Direct cytostatic effects of propranolol were independent of apoptosis induction and were associated with reduced mitosis, G0/G1 cell cycle arrest and a significant down-regulation of cell cycle regulator Cyclin D1. Moreover, colony formation, 3D spheroid growth, cell chemotaxis and capillary-like tube formation were drastically impaired after propranolol treatment. Interestingly, anti-migratory activity of β-blocker was associated with altered actin cytoskeleton dynamics. In vivo, propranolol treatment (10 mg/kg/day i.p.) reduced the early angiogenic response triggered by MG-63 cells in nude mice. Synergistic effects were observed in vitro after combining propranolol with chemotherapeutic agent cisplatin. Sustained administration of propranolol (10 mg/kg/day i.p., five days a week), alone and especially in addition to low-dose metronomic cisplatin (2 mg/kg/day i.p., three times a week), markedly reduced xenograft progression. After histological analysis, propranolol and cisplatin combination resulted in low tumor mitotic index and increased tumor necrosis. β-blockade using propranolol seems to be an achievable and cost-effective therapeutic approach to modulate osteosarcoma aggressiveness. Further translational studies of propranolol repurposing in osteosarcoma are warranted.

## Introduction

Osteosarcoma (OSA) is the most prevalent primary bone tumor, affecting mostly pediatric and juvenile age‐group patients^1^. In addition to surgical removal of primary and secondary lesions, management of OSA involves adjuvant administration of chemotherapy, including methotrexate, doxorubicin and cisplatin (CDDP)^2^. However, chemotherapy effectiveness in OSA patients is restricted by drug resistance development by tumor cells, lack of selectivity and high toxicity^3^. Taking this into account, there is an urgent need to develop novel, cost-effective and selective therapeutic alternatives for the treatment of aggressive OSA, putting emphasis on relevant biological events that rule disease progression.

Drug repurposing in oncology is defined as the implementation of already-approved drugs for other medical applications to treat cancer^4^. In comparison to de novo drug discovery, both development risks and costs, as well as safety-related failure, are reduced with this approach because such drugs have well-known pharmacokinetic/pharmacodynamic profiles, and pharmacovigilance for adverse effects is largely accessible^5^. Moreover, in the search for greater therapeutic benefits, repurposed drugs are often combined with metronomic chemotherapy, consisting of frequent administration of low-dose chemotherapy, with no sustained drug-free breaks. This treatment strategy stands as an interesting therapeutic approach as its reduced costs and fewer infrastructure needs can be easily adapted in financially-constrained health care systems^6^.

Propranolol (PPN) is a non-selective β-adrenergic receptor (ADRB) antagonist that was originally used for the treatment of hypertension and other cardiovascular conditions^7^. Over the last years, PPN has also demonstrated potent anticancer effects in a wide variety of oncological settings. After initial clinical retrospective observations and subsequent clinical trials, this β-blocker was first repurposed for the treatment of infantile hemangioma, a common vascular tumor of infancy^8^. Preclinical studies showed that ADRB type 1 (ADRB1) and 2 (ADRB2) blockade using PPN results in cell cycle arrest, apoptosis and proliferation reduction of breast, melanoma and liver tumor cells, among others, mainly by disrupting mitogenic signaling cascades involving protein kinase B (PKB) and mitogen activated protein kinase (MAPK)^9–12^. PPN was also capable of reversing protumoral effects triggered by stress hormones after surgery, increasing immune function and impairing metastasis^7^. Assessment of PPN anticancer properties has recently reached clinical stages for angiosarcoma and other tumor types such as melanoma, exploring its benefits as a surgical adjuvant or in combination with chemotherapy and different targeted therapies^13,14^.

Although sympathetic nervous system (SNS) activation and catecholamine-triggered signaling pathways are deeply involved in cancer initiation and growth^15^, little is known about the specific role of β-adrenergic signaling in OSA progression. Interestingly, expression of catecholamines and their receptors was previously reported in multiple human and canine OSA models^16,17^. Moreover, norepinephrine and β-adrenergic receptors were also detected in OSA clinical samples, showing increased expression levels in comparison to matched non-oncological healthy bone^18^, making the exploration of β-blocker PPN in OSA an appealing niche for further studies. Considering the unsatisfied clinical needs of OSA and the multiple reported benefits of PPN as a broad antitumor agent, the aim of the present study was to evaluate the in vitro*/*in vivo antitumoral activity of PPN in OSA, as a monotherapy or in combination with metronomic chemotherapy.

## Results

**Figure 1:**
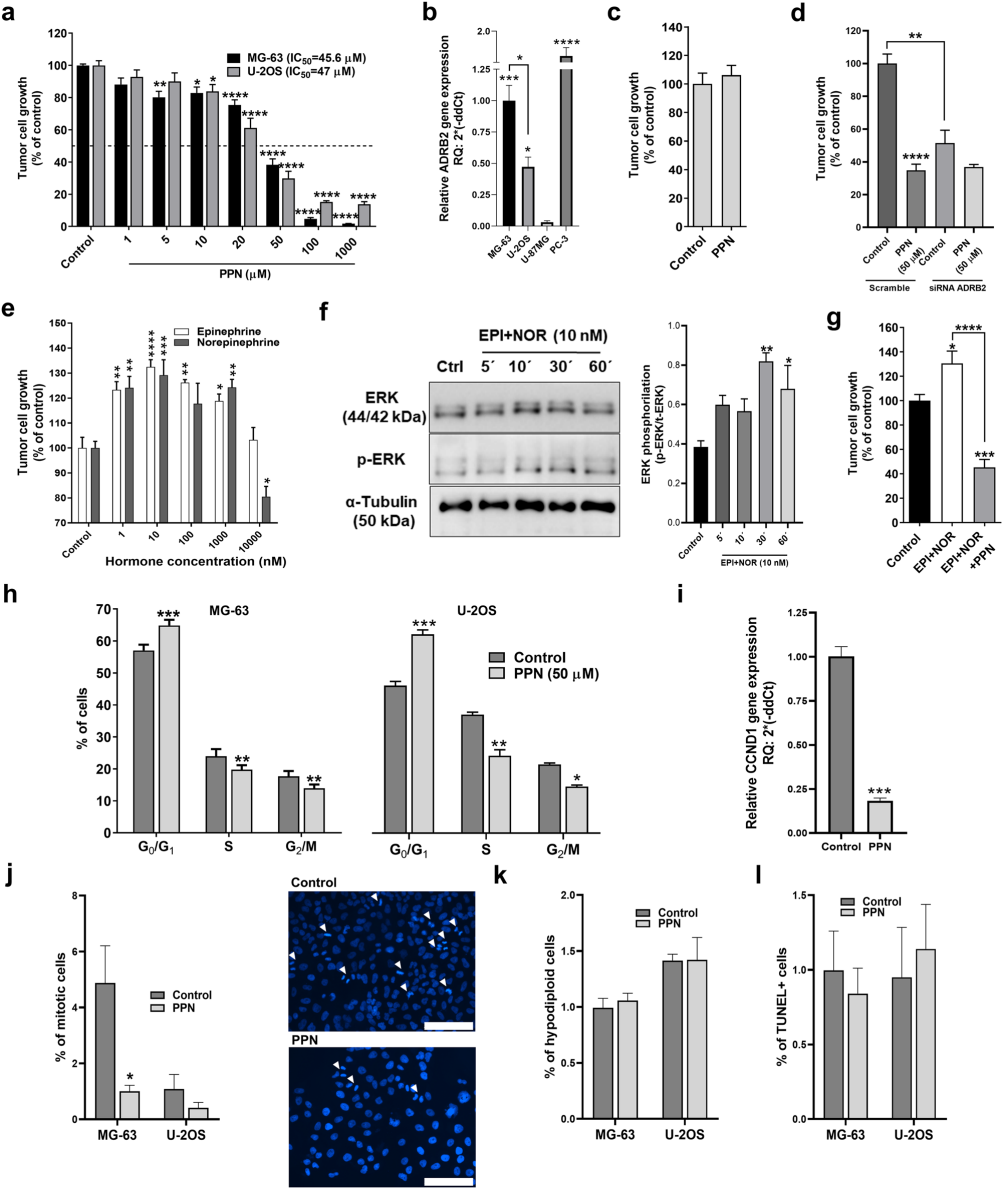
Direct cytostatic activity of β-blocker propranolol on osteosarcoma cells. Tumor cell growth was evaluated on log-phase growing osteosarcoma (OSA) cells and measured by crystal violet staining after a 72 h exposure to the tested compound. (**a**) Inhibition of cell growth by propranolol (PPN, 1–1000 µM) on MG-63 and U-2OS cell cultures, and calculation of IC_50_ values. Statistical significance for PPN in both cell lines was achieved with drug concentrations ≥ than 10 µM. (**b**) Expression of β2-adrenergic receptor (ADRB2) by OSA cells. ADRB2 mRNA expression levels were evaluated by qRT-PCR in MG-63 and U-2OS OSA cells, human prostate PC-3 cells (positive control expression) and human glioma U-87MG cells (negative control expression). (**c**) Absence of non-specific effect of PPN in ADRB2-negative U-87MG cells. (**d**) MG-63 cells were transiently transfected with ADRB2-targeting siRNA (siRNA ADRB2) or non-targeting control siRNA (Scramble). Cells were treated for 72 h with PPN (50 µM) and cellular growth was measured using the crystal violet assay. (**e**) Stimulation of OSA cell growth by ADRBs agonists epinephrine (EPI) and norepinephrine (NOR) (1–10,000 nM). (**f**) Western blotting was performed to analyze the impact of EPI and NOR treatment on ERK1/2 signaling pathway in MG-63 cells. Original blots are presented in Fig. S1. (**g**) Blockage of catecholamines EPI and NOR growth-stimulating effect by coincubation with low dose PPN (10 µM) in MG-63 cells. (**h**) Cell cycle phase distribution for OSA cells, MG-63 and U-2OS, treated with PPN (50 µM) during 24 h obtained by flow cytometry analysis. (**i**) Gene expression analysis of cyclin D1 (CCND1) by qRT-PCR in MG-63 cells after 24 h treatment with PPN at 50 µM. (**j**) Calculation of MG-63 and U-2OS cells in vitro mitotic index was performed on DAPI-stained OSA cell cultures using fluorescence microscopy. Mitotic cells were identified after 48 h PPN treatment in ten HPF of ×400 magnification per experimental group. Representative fluorescent images of vehicle (upper) or PPN (50 µM) treated (lower) OSA cultures showing different mitotic bodies (White arrows). Scale bar = 100 µm. (**k**) MG-63 and U-2OS cell apoptosis was evaluated as the percentage of cells in the sub-G_0_/G_1_-phase, according to their hypodiploid DNA content. (**l**) % of apoptotic cells in 48 h-treated MG-63 and U-2OS cell cultures were also assessed by TUNEL labeling. Data are presented as mean ± SEM and are representative of at least two or three independent experiments. *p < 0.05, **p < 0.01, ***p < 0.001 and ****p < 0.0001. ANOVA followed by Tukey’s test for (a), (b), (d), (e) and (g), ANOVA followed by Dunnet’s test for (f), unpaired *t* test for (c), (h), (i), (j) and (k), and χ^2^ test for (l).

First, in order to evaluate in vitro OSA sensitivity to PPN, semiconfluent monolayers of rapidly growing MG-63 and U-2OS cells were exposed to tested compound using a concentration range between 1 µM and 1 mM, based on previously reported preclinical studies^10–12^. After a 72 h exposure to PPN, we observed a dose-dependent cell growth inhibition in both tested cell lines obtaining IC_50_ values of 45.6 and 47 µM, respectively (Fig. 1a). It is worth noting that this cytostatic activity was achieved in the absence of β-AR endogenous agonists. ADRB2 expression was confirmed in MG-63 and U-2OS cells by qRT-PCR, with MG-63 cells yielding higher gene expression values (Fig. 1b). Human prostate PC-3 cells, known to overexpress ADRB2, were used as an internal positive control^19^. In addition, human glioma U-87MG cells were used as a negative control^20^. In order to evaluate nonspecific effects of PPN, we exposed ADRB2-negative U-87MG cells to PPN and assessed its impact on cellular growth. Interestingly, no gross off-target cytostatic activity was observed in U-87MG cells after a 72 h exposure to PPN at 50 µM (≈ IC_50_ in responsive cells) (Fig. 1c). MG-63 cells were transiently transfected with ADRB2-targeting or non-targeting scrambled siRNAs, and treated with PPN in order to confirm ADRB2-mediated antiproliferative effects. First, ADRB2 knock-down caused a significant attenuation of PPN in vitro effect. When compared to scrambled-transfected cells, ADRB2 depletion resulted in a relative 54% reduction of PPN cytostatic activity (Fig. 1d). Furthermore, growth capacity of MG-63 cells transfected with ADRB2 siRNA was severely affected, showing a cell growth reduction of 48.6% in comparison to the scramble group (Fig. 1d).

ADRB2-positive MG-63 cells were then incubated with epinephrine (EPI) and norepinephrine (NOR) with the aim of evaluating their effect on cellular growth. Both catecholamines are direct-acting and non-selective α and β-adrenergic agonists. After a 72 h stimulation, EPI and NOR significantly increased OSA cell growth, showing a maximum growth-stimulatory effect at 10 nM (Fig. 1e). Interestingly, incubation with selective ADRB1 agonist dobutamine had no impact on MG-63 cell growth rate (Fig. S2). ADRB2 is a Gs-coupled receptor known to activate downstream mitogen-activated protein kinase (MAPK)-associated signaling pathways^21,22^. In order to further explore catecholamine stimulatory activity on OSA growth, we examined extracellular signal-regulated kinase (ERK) phosphorylation in MG-63 cells after ADRB2 activation by EPI and NOR (10 nM). As a result a significant increase in the phosphorylated ERK/total ERK ratio was observed after 30 and 60 min stimulation with catecholamines (Fig. 1f). As shown in Fig. 1g, addition of PPN (10 µM) to EPI plus NOR (both at a final concentration of 10 nM) was capable of completely blocking the growth-stimulating activity of catecholamines. Interestingly, growth inhibition was greater in OSA cell cultures exposed to PPN and catecholamines, in comparison to PPN alone (18% versus 54%, respectively). Similar findings were previously reported^23^, and despite the fact that the specific mechanisms remain unclear, this results could be linked to co-expression of α-2A adrenoceptor (ADRA2A) in MG-63 cells^16^, which, in contrast to ADRB2 receptor, is associated with inhibitory responses in noradrenergic neurons^24^ and also with antitumor signaling in cancer cells^25^.

Next, with the aim of exploring potential mechanisms associated with cell growth inhibition by PPN we conducted cell cycle phase distribution analysis in OSA cells. A significant arrest in the G_0_/G_1_ cell cycle phase and subsequent decline in both S and G_2_/M phases were observed in both MG-63 and U-2OS cells following a 24 h treatment with PPN (50 µM), as evaluated by flow cytometry studies (Fig. 1h). Cyclin D1 (CCND1) serves as a central regulator for cell cycle progression, and its overexpression results in dysregulated cyclin-dependent kinases activity, rapid cell growth under conditions of restricted mitogenic signaling, and ultimately, tumor growth^26^. As shown in (Fig. 1i), cell cycle blockade was associated with a significant reduction in CCND1 expression, as its relative gene expression values plummeted by 80% in PPN-treated MG-63 cells in comparison to vehicle-treated cells. To discard any short-term direct cytotoxicity of PPN on such treatment schedule, semiconfluent OSA cell cultures exposed to PPN were assessed by the trypan blue dye exclusion assay, showing that PPN at ≈ IC_20_ and IC_50_ concentrations had no effect on cell viability (data not shown). Interference of cell cycle progression after β-blockage was further confirmed by in vitro mitotic index calculation in MG-63 and U-2OS cultures exposed to DAPI for DNA and chromosome staining. Mitotic bodies that were included for quantification were representative of prophase through telophase. PPN caused a significant inhibition in the % of mitotic cells in MG-63 cultures (Fig. 1j). In U-2OS cultures, which exhibit notorious lower mitotic rates, a reduction of approximately 60% in the number of mitosis was also observed, but no statistical significance was achieved. Despite the large amount of preclinical/clinical data reporting its pro-apoptotic effects on malignant cells^9,11^, PPN at a concentration ≈ IC_50_ was not capable of inducing apoptosis in MG-63 or U-2OS cells, as evaluated by quantification of hypodiploid sub-G_0_/G_1_ cell populations and TUNEL labeling (Fig. 1k and l).

**Figure 2:**
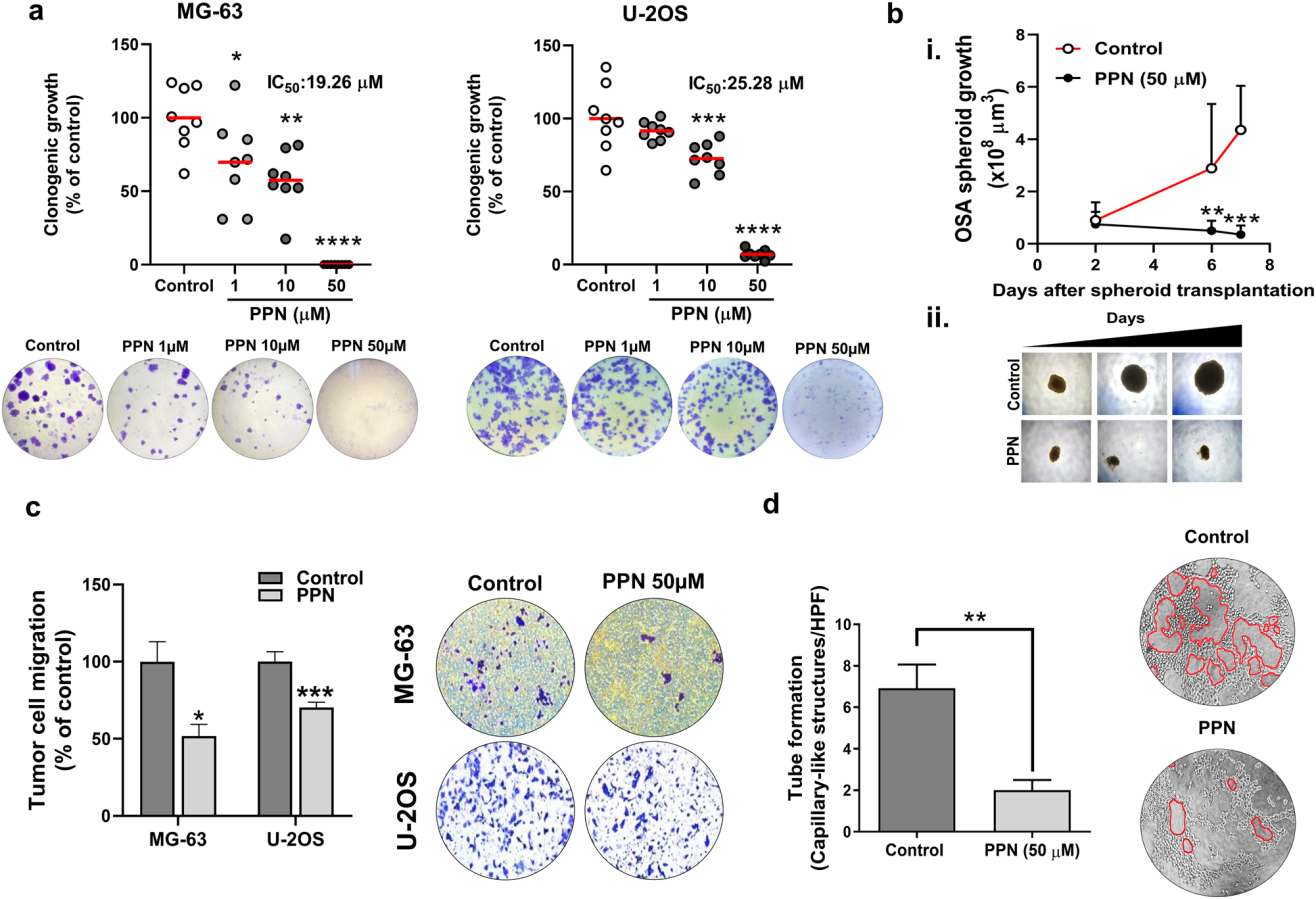
Propranolol abrogates colony formation, three-dimensional spheroid growth, cell migration and tube formation in osteosarcoma. (**a**) Number of osteosarcoma (OSA) colonies per well quadrant was quantified after a 7 day incubation with propranolol (PPN, 1–50 µM). Representative microphotographs of MG-63 and U-2OS clonogenic cultures (bottom) in control and PPN 1, 10 and 50 µM groups (left to right) are depicted. (**b**) PPN completely impairs growth of fully formed OSA spheroids. (i) OSA spheroid volume was assessed as a direct parameter of spheroid growth during the following week. (ii) Representative images of OSA spheroids at days 2, 6 and 7 (left to right). ×40 final magnification. (**c**) Inhibition of MG-63 and U-2OS cell chemotaxis after a 16 h treatment using PPN at 50 µM. Representative pictures of tumor cells on the bottom of the *Transwell* inserts (bottom) corresponding to the control or PPN group (left and right, respectively) are shown. (**d**) Inhibition of capillary-morphogenesis on a Matrigel-coated substrate by MG-63 cells after 16 h treatment with PPN 50 µM. Representative images of capillary-like tubular structures (identified in red lining) in different experimental conditions (×100 magnification). *p < 0.05, **p < 0.01, ***p < 0.001 and ****p < 0.0001. ANOVA followed by Tukey's test for (**a**), unpaired two-sided *t* test for (**b**) and (**c**) and Mann–Whitney test for (**d**).

Early metastatic spread and recurrences after tumor removal are strongly related to poor prognosis in OSA^27^. Considering that tumor cell migration, colony establishment and microtumor outgrowth are all hallmarks of the early stages of metastatic progression, we tested the effect of β-blocker PPN on the establishment and progression of 2D tumor colonies, 3D spheroid growth, OSA cell chemotaxis and vascular morphogenesis. Clonogenic growth of OSA cells was severely suppressed after 7 days of incubation with 1, 10 and 50 µM of PPN, reducing colony forming capacity by 31, 46 and 100% in MG-63 cells and 8, 27 and 96% in U-2OS cells, respectively, in comparison to vehicle-treated cells (Fig. 2a) (IC_50_ = 19.26 and 25.28 µM for MG-63 and U-2OS cell lines, respectively). The latter IC_50_ values are substantially lower than those previously observed in cell growth assays (approximately 45 µM for both tested cell lines), showing that OSA cells are particularly sensitive to prolonged PPN exposure, especially during colony establishment and outgrowth. Additionally, metabolic activity of MG-63 colonies was assessed using the MTS assay in which exposure to PPN (1, 10 and 50 µM) reduced proliferation by 25, 36 and 94%, depending on drug concentration (Fig. S3). As above-mentioned, anti-OSA activity was also evaluated on established multicellular spheroids during a 7 day treatment period. MG-63 cells were chosen for their capacity of forming homogeneous and rapidly-growing spheroids. In contrast to control spheroids which grew until reaching a sixfold increase in volume, growth of PPN-treated spheroids was completely blocked (Fig. 2b, i and ii), as observed in representative photographs. Chemotaxis of MG-63 and U-2OS cells after an overnight exposure to PPN (50 µM) was also significantly impaired, causing a cell migration reduction of 48 and 39%, respectively (Fig. 2c). Capillary-like tube formation on Matrigel-coated substrates was also evaluated in MG-63 cells as a measure of vasculogenic activity. This phenomenon, also known as vascular mimicry, is highly dependent on enhanced cell motility and migratory properties^28^, and is associated with worse prognosis in OSA^29^. As shown in Fig. 2d, PPN (50 µM) treatment was capable of effectively impairing tube formation in MG-63 cells, reducing the number of capillary-like structures by nearly 70%.

**Figure 3:**
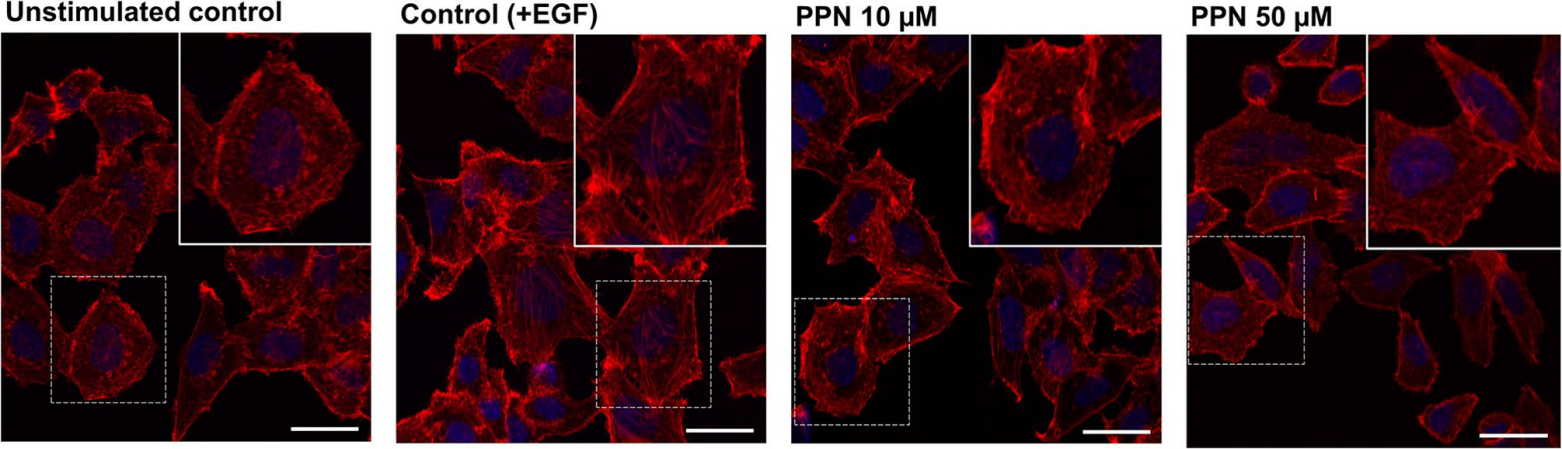
Propranolol modulates epidermal growth factor-induced actin cytoskeleton reorganization and stress fiber formation in osteosarcoma cells. Representative images of MG-63 cells stained with AlexaFluor555-conjugated phalloidin (red) after exposure to saline vehicle (unstimulated control), epidermal growth factor (EGF) (control), and PPN 10 or 50 µM, in addition to EGF stimulation. Nuclei were counterstained with DAPI (blue). Actin fluorescent staining in MG-63 cells grown was assessed by confocal laser scanning microscopy. Insets contain enlarged representative cells indicated by dashed squares. (×630 magnification. Scale bar = 20 µm).

It is well established that epidermal growth factor (EGF) induces cytoskeleton reorganization and cell migration in OSA cells^30,31^. Moreover, EGF receptor (EGFR), and its related downstream pathways, are particularly overexpressed in the MG-63 cell line in contrast to other OSA cell lines^31^. Therefore, in order to evaluate whether PPN could interfere with EGF-induced actin polymerization in OSA cells characterized with high functional levels of EGFR, we treated serum-starved MG-63 cells with PPN for 1 h and then stimulated the cells for 20 min with EGF (see "Methods" section). As shown in Fig. 3, stimulation with EGF efficiently induced actin polymerization in control cells. However, in the presence of PPN (10 and 50 µM), EGF-induced actin reorganization and stress fiber formation was significantly blocked (Figs. 3 and S4A). Additionally, PPN treatment was associated with increased tumor cell rounding and a reduction in cell spreading (Figs. 3 and S4a,c).

**Figure 4:**
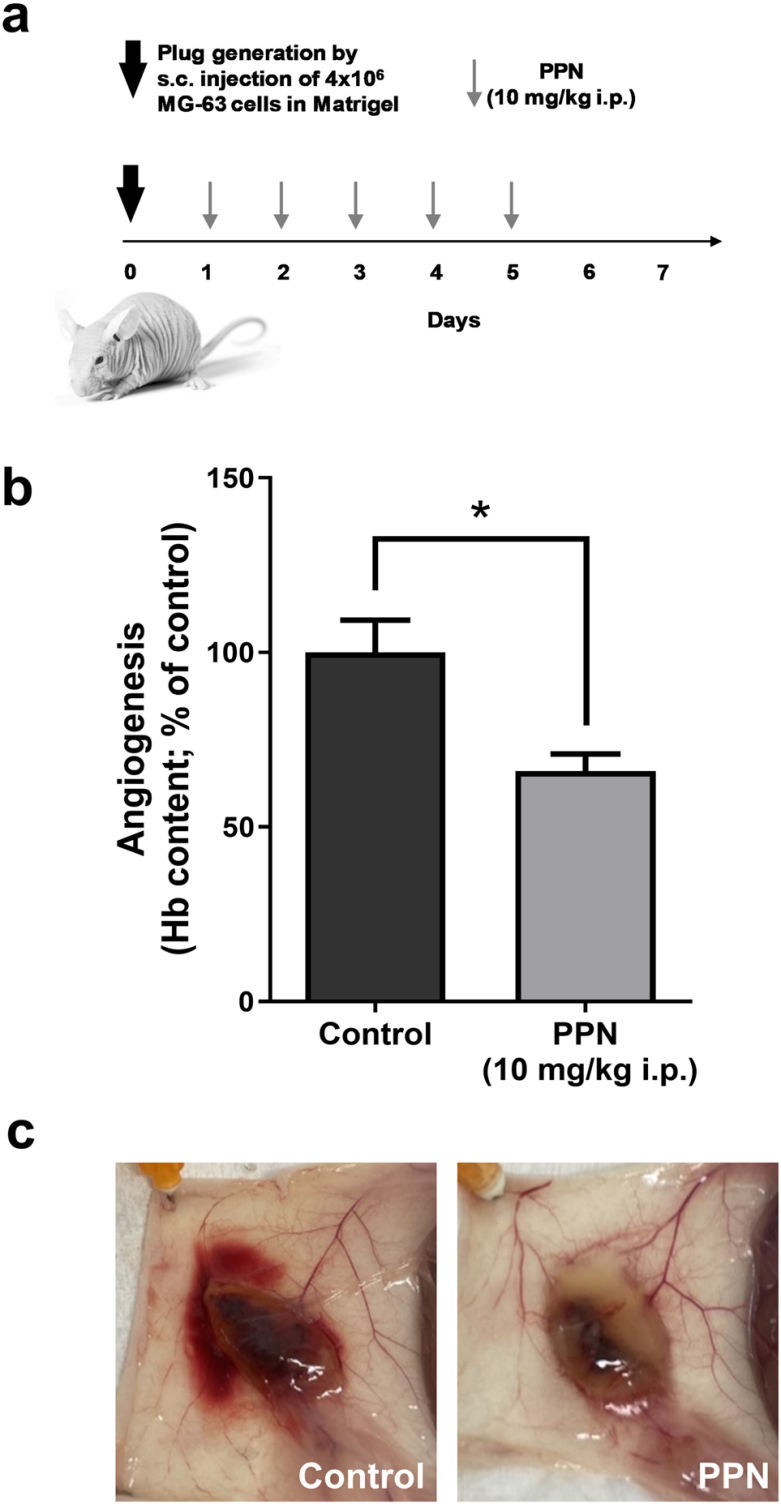
Propranolol treatment inhibits osteosarcoma-driven angiogenesis. Impact of propranolol (PPN) administration (10 mg/kg/day, i.p.) on MG-63-induced angiogenesis was assessed using a modified-Matrigel plug assay in nude mice. (**a**) Experimental design and PPN dosing. (**b**) Early angiogenic response was assessed by quantification of hemoglobin content in the recovered Matrigel plugs using the Drabkin’s method (see "Methods" section). (**c**) Representative photographs of subcutaneous vascularized implants in vehicle or PPN-treated animals. Five animals per experimental group. Data are presented as mean ± SEM. *p < 0.05, unpaired *t* test.

In OSA, as in many other aggressive cancers, angiogenesis and overexpression of pro-angiogenic markers correlate with disease progression^32^. In this setting, and considering the large amount of data that supports the angiostatic profile of PPN, we evaluated the effect of PPN administration on in vivo OSA-induced angiogenesis (Fig. 4). Subcutaneous plugs containing a commercial preparation of purified basement membrane matrix in addition to MG-63 cells and heparin were generated in athymic mice, and early vascular response was assessed by hemoglobin quantification after 1 week of plug implantation (see "Methods" section). Animals received PPN at a dose of 10 mg/kg/day i.p., or vehicle, for five consecutive days. As observed in Fig. 4, PPN administration was capable of impairing OSA cell-induced early angiogenic response, even after a one-week treatment, reducing by 34% the hemoglobin content in s.c. plugs, in contrast with saline vehicle-treated animals (Fig. 4a,b).

**Figure 5:**
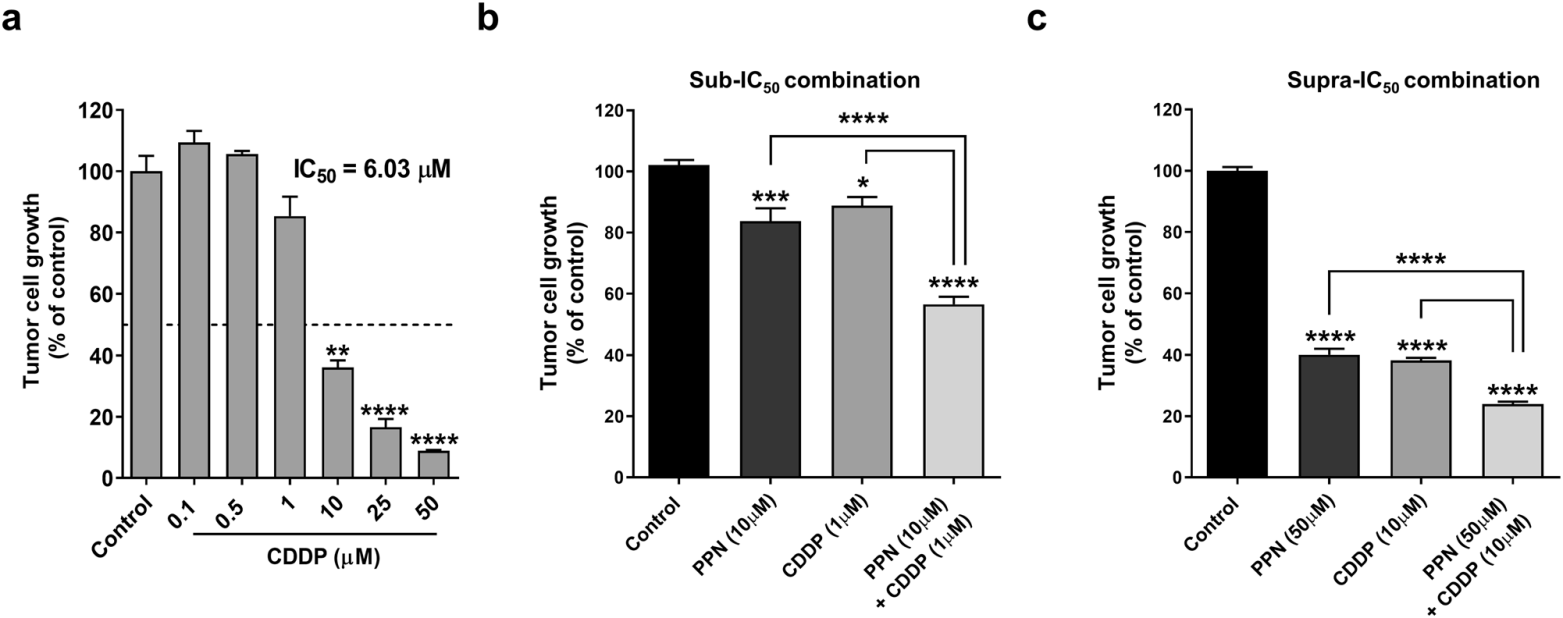
Cooperative growth-inhibiting activity of propranolol in combination with cisplatin in osteosarcoma cells. Tumor cell growth after propranolol (PPN) plus cisplatin (CDDP) combined treatment was evaluated on log-phase growing MG-63 cells and measured by crystal violet staining. (**a**) Impact of CDDP (0.1–50 μM) on OSA cell survival and IC_50_ calculation after a 72 h exposure to tested compounds. Statistical significance (*p < 0.05) in growth inhibition by CDDP was achieved with drug concentrations ≥ 10 μM. (**b**) and (**c**), potential cooperative effects between PPN and CDDP were assessed by combining different sub-IC_50_ or supra-IC_50_ concentrations of tested compounds. Data are presented as mean ± SEM and are representative of at least two or three independent experiments. *p < 0.05, ***p < 0.001, ****p < 0.0001. ANOVA followed by Tukey's test for (a), (b) and (c).

DNA-damaging agent CDDP remains as a cornerstone in the treatment of OSA^2^. With the aim of evaluating potential therapeutic benefits after addition of PPN to metronomic chemotherapy, we first conducted in vitro combination studies exploring the cytostatic effects of PPN plus CDDP (see "Methods" section). After calculating IC_50_ value for CDDP as a monotherapy (Fig. 5a), sub- and supra-IC_50_ concentrations of PPN and CDDP were defined for combination assays. Synergy (CI < 1) was observed in both low- and high-concentration combination scenarios after addition of PPN to CDDP (Fig. 5b,c), obtaining combination indexes of 0.36 and 0.55 for the PPN 10 µM + CDDP 1 µM, and PPN 50 µM + CDDP 10 µM combinations, respectively. Corresponding dose-reduction indexes were found as 3.96 and 1.87, respectively.

**Figure 6:**
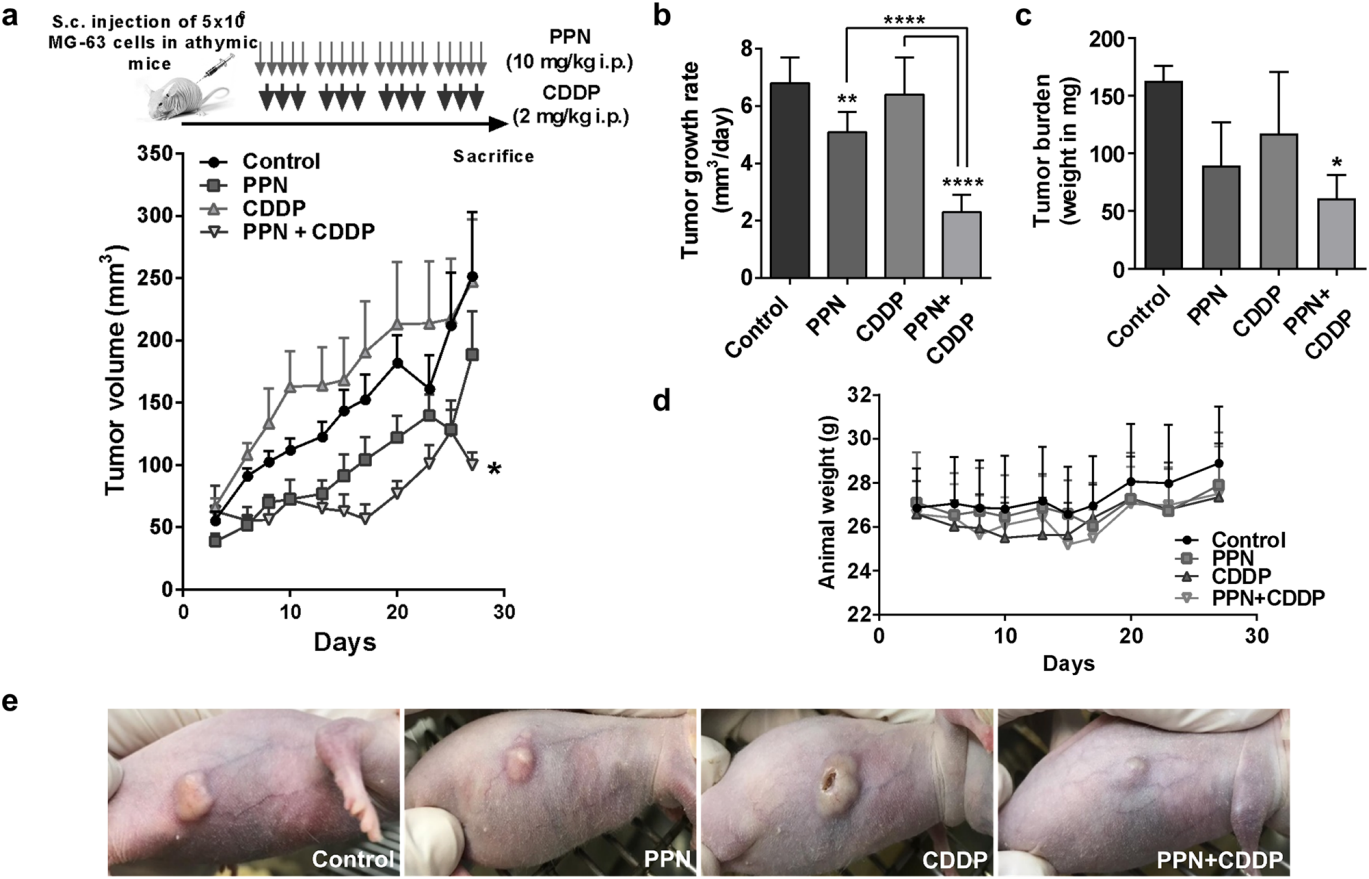
Propranolol in combination with low-dose metronomic cisplatin inhibits osteosarcoma tumor growth without overt signs of toxicity. (**a**) Experimental design used to evaluate in vivo effects of propranolol (PPN, 10 mg/kg i.p.) plus cisplatin (CDDP, 2 mg/kg i.p.) on tumor progression of MG-63 xenografts growing in athymic mice (Top). Curves represent mean tumor volumes of mice receiving saline vehicle (control), PPN, CDDP or PPN plus CDDP over time (bottom). 5 animals per experimental group. (**b**) Aggressiveness of osteosarcoma (OSA) xenografts was additionally assessed by calculating tumor growth rates between days 3 and 27 of protocol. (**c**) Final tumor burden was evaluated by weighing resected OSA primary lesions after necropsy. (**d**) Body weight of animals bearing MG-63 xenografts belonging to different experimental groups throughout the protocol. (**e**) Representative photographs of nude mice bearing OSA xenografts belonging to different experimental groups at day 30 are shown. Data are presented as mean ± SEM. *p < 0.05, **p < 0.01, ****p < 0.0001. ANOVA followed by Tukey’s test.

Taking these results into consideration, the anti-OSA activity of PPN in combination with CDDP was further evaluated in vivo on MG-63 xenografts (Fig. 6a). Animals received i.p. PPN at a dose of 10 mg/kg in a 5-day-on/2-day-off schedule, as a monotherapy or in combination with low-dose metronomic CDDP using 2 mg/kg i.p. three times per week until the end of the protocol. This scheduling differs with conventional dosing schemes which rely on few cycles of high-dose chemotherapy which are often interrupted by drug-free intervals and detoxification protocols^33–35^ (see Methods section). Treatment with PPN in addition to sustained low-dose CDDP significantly reduced OSA xenograft progression, slowing tumor growth rate by 70% in comparison to vehicle-treated mice and significantly improving the anti-OSA activity of PPN or CDDP alone (Fig. 6b). PPN treatment as a single agent was also capable of reducing tumor growth, causing a less profound reduction of about 20%. Therapeutic benefits of PPN plus CDDP were confirmed after measuring final tumor burden, where mean tumor weight was reported as 146.7 ± 38.9 mg (mean ± SD) in the control group in comparison to 53 ± 23.8 mg in the PPN plus CDDP group (Fig. 6c). It is important to highlight that, despite observing significant effects in terms of tumor growth reduction, all tumors progressed on treatment, regardless of therapeutic intervention. All treatments were well tolerated as no significant changes in animal body weight, food or water consumption were observed throughout the protocol (Fig. 6d). Representative OSA tumor-bearing mice from different experimental groups are depicted in Fig. 6e.

**Figure 7:**
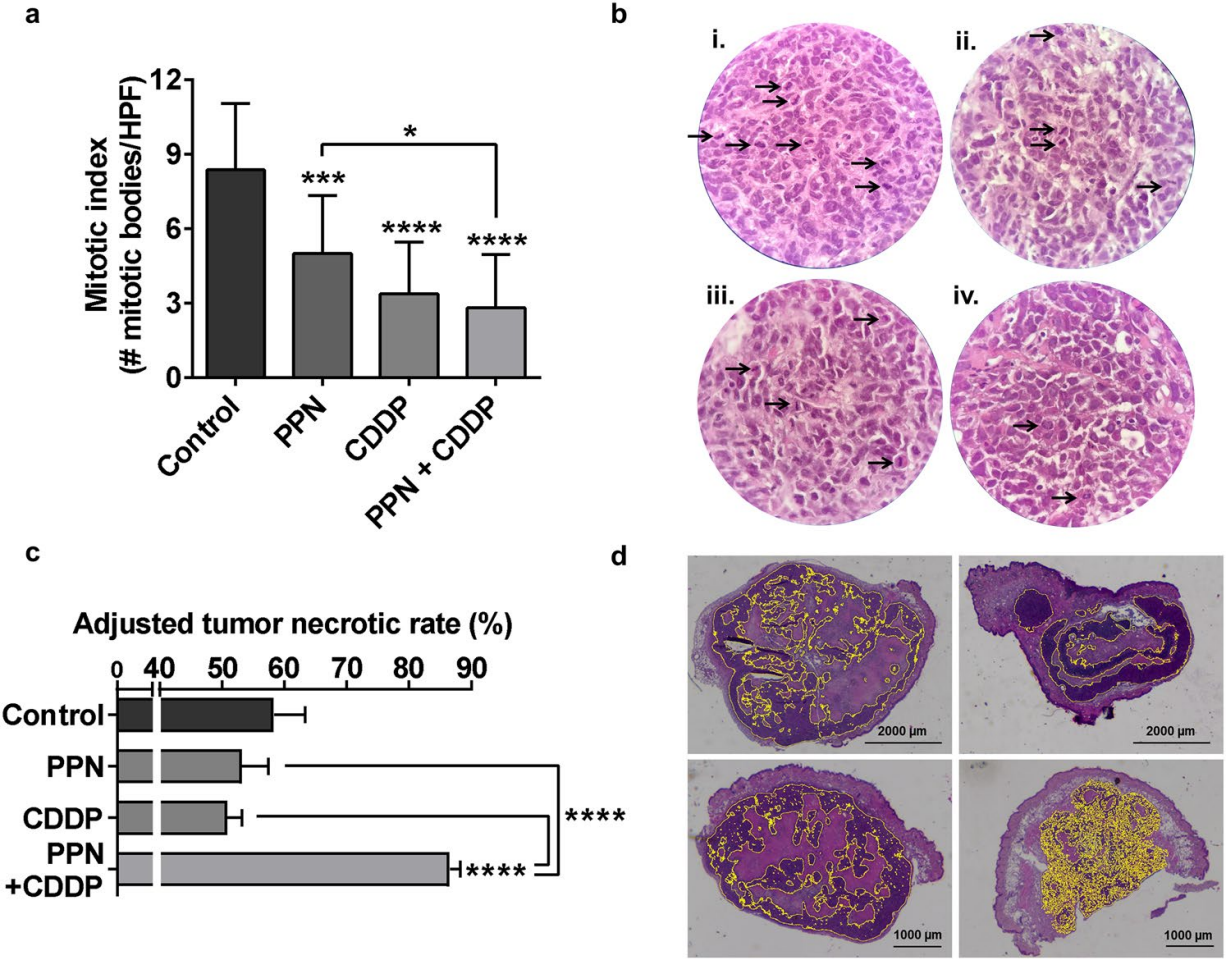
Histopathological assessment of mitotic index and necrosis in osteosarcoma tumors after treatment. (**a**) Antimitotic activity of propranolol (PPN) in combination with metronomic cisplatin (CDDP) on osteosarcoma (OSA) xenografts. Quantification of mitotic index in OSA tumor sections from different experimental groups was expressed as the number of mitotic bodies per HPF. (**b**) Representative images of H&E stained tumor slides belonging to animals treated with i) vehicle, ii) PPN, iii) CDDP or iv) PPN plus CDDP. Arrows show tumor cell mitotic bodies (×400 magnification). (**c**) % of tumor necrosis in OSA samples was adjusted to tumor volume variation as a response to specific treatments, and was expressed as adjusted tumor necrotic rates. (**d**) Representative digitally-stitched images of complete tumor sections from vehicle (upper left), PPN (upper right), CDDP (lower left) and PPN plus CDDP (lower right) treatment groups. Yellow lines in tumor slides mark highly basophilic tissue regions belonging to viable malignant tissue. Scale bars = 1000 or 2000 µm. Data are presented as mean ± SEM. *p < 0.05, ***p < 0.001, ****p < 0.0001. ANOVA followed by Tukey’s test.

Given that high mitotic counts are associated to OSA aggressiveness and poor prognosis^36^, mitotic index was assessed in resected tumors obtaining values of 8.3 ± 2.7, 5 ± 2.3, 3.36 ± 2.1 and 2.81 ± 2.2 (mean ± SD) mitotic bodies per high power field for control, PPN, CDDP and PPN + CDDP groups, respectively (Fig. 7a,b). As shown in Fig. 6a, although all tested therapies achieved significant reductions on mitotic counts, sustained PPN + CDDP therapy yielded the most potent effect (67% inhibition in comparison to vehicle-treated group). Interestingly, despite not being reflected on its effect on OSA growth, sustained low-dose CDDP treatment was linked to an evident reduction in the proliferating status of OSA cells within the tumor, quite equivalent to combined therapy. Considering that assessment of tumor necrosis in OSA has become a relevant tool for evaluating response to therapy^37,38^, adjusted tumor necrotic rates (ATNR) were calculated for all experimental groups (Fig. 7c) (see Methods section). Only PPN plus CDDP-based therapy was capable of significantly enhancing ATNR in comparison to vehicle-treated animals, from 58.32 ± 5.04 to 81.74 ± 2.25%, respectively. Besides a marked increase in ATNR, tumors belonging to PPN + CDDP-treated animals displayed a peculiar diffuse and rather centered pattern of viable tumor tissue in contrast to control, PPN or CDDP groups, where viable tumor tissue areas were found as large and scattered basophilic regions, interrupted by vast areas of necrotic tissue (Fig. 7d).

## Discussion

To the best of our knowledge this is the first study to report the therapeutic benefits of non-selective β-adrenergic antagonist PPN in combination with chemotherapy in human OSA experimental models. Exposure of OSA cells to PPN was associated with reduced cell growth and mitosis, G_0_/G_1_ cell cycle arrest and impairment of 2D/3D tumor colony formation and growth, among other effects. Similar IC_50_ values of approximately 50 µM were obtained for PPN on both ADRB2-expressing MG-63 and U-2OS cell lines, confirming equivalent sensitivities for the compound. After analyzing other previously published studies, obtained IC_50_ values were equivalent or, in some cases, substantially lower in comparison to other aggressive pediatric tumor types (IC_50_ ranging from 114 to > 200 µM)^11,12^. However, a large number of recently reported preclinical studies focused specifically on β-adrenergic signaling consistently used a 50 µM concentration for characterizing the antineoplastic profiles and mechanisms of action of both non-selective and selective β-blockers in a wide variety of tumor types^22,39–41^.

PPN direct cytostatic activity showed to be ADRB2-dependent. Silencing of ADRB2 using targeting siRNA significantly attenuated the inhibitory effects of PPN on OSA cell growth compared to scrambled-transfected control cells. Nonetheless, considering that the attenuation of the effect was not total, other off-target or non-specific alternative mechanisms cannot be completely ruled out, especially when PPN is used at high concentrations. Also in this regard, the exploration and characterization of other β-blockers in OSA, especially selective β antagonists (i.e. Metoprolol for ADRB1 or ICI 118,551 for ADRB2) would be highly interesting from a mechanistically point of view. However, a large number of preclinical and clinical studies demonstrated that non-selective β-AR antagonists, such as PPN or atenolol, are superior to selective β-AR antagonists at inhibiting tumor progression, cell survival and improving clinical outcome^13,42–44^.

OSA cell growth seems to be influenced by catecholamines. Exposure to low concentrations of non-selective adrenergic agonists EPI and NOR resulted in increased OSA growth and activation of the ERK1/2-MAPK signaling pathway, in accordance to previously reported data^21,22^. Besides confirming that exposure of ADRB2-positive OSA cells to endogenous catecholamines results in growth stimulation, here we show that MG-63 growth inhibition was greater in cell cultures exposed to PPN and catecholamines in contrast to PPN alone. The same result was observed in a previous study published by Coelho et al*.* in which adrenergic signaling was evaluated in colorectal cancer cells^23^. This happened with non-selective β-blockers PPN and carvedilol, but not with specific ADRB1 and ADRA1 antagonists. Despite the specific mechanism being unclear, we hypothesize that the enhanced growth-inhibiting effect of PPN plus catecholamines, in comparison to PPN alone, could be related to the fact that MG-63 cells co-express ADRA2A in addition to ADRB2^16^. ADRA2A acts as a presynaptic autoinhibitory receptor in noradrenergic neurons^24^, but is also expressed in some tumor types^25,45^. Different authors have demonstrated that high expression of ADRA2A was associated with a better prognosis, and its overexpression was capable of suppressing tumor invasion and growth through inhibition of the PI3K/Akt/mTOR pathway^25,45^. Therefore, as both ADRA2A and ADRB2 receptors are co-expressed in MG-63 cells, and ADRA2A is associated to antiproliferative signaling upon stimulation in malignant cells, ADRB2 blockade by PPN and ADRA2A activation by present catecholamines could collaborate into a greater tumor cell growth inhibition of MG-63 cells.

Interestingly, no significant proapoptotic activity of PPN on OSA cells was observed at 50 µM. However, it is worth noting that apoptosis induction in other preclinical studies using PPN on different tumor models was achieved using concentrations in the range of 100–300 µM^9,11,12^. Exploring such concentrations goes beyond the scope of the present work given that, according to early pharmacokinetics reports, achievable plasma concentrations of PPN are in the low µM range^46,47^.

Growth inhibition and impairment of colony formation in both MG-63 and U-2OS cells were associated with cell cycle blockade in the G_0_/G_1_ phase, and a significant suppression of cell cycle regulator cyclin D1 (CCND1). These results are in accordance with previous data reported by Montoya et al., who identified cyclin D1 (among a large panel of cyclin proteins) as a key mediator of PPN inhibitory effect on mitogenic potential of breast cancer cells^9^. PPN cytostatic activity in ADRB-expressing malignant cells was also previously associated to the down-regulation of the AKT/mammalian target of rapamycin (mTOR) and the MEK/ERK1/2 MAPK signaling pathways^9–11^. Interestingly, with the aim of shedding some light into the largely unknown molecular landscape of OSA, genomic and transcriptomic studies have recently identified frequently mutated genes and copy number alterations in OSA samples^48,49^. Besides confirming TP53, RB1 and MYC as driver genes, it was reported that PI3K/AKT/mTOR and MAPK pathways are heavily altered in OSA and are central players in disease initiation and progression. PPN reduced OSA cell migratory capacity and tubule formation, and impairment of chemotaxis was associated with altered cytoskeleton dynamics and reduction of EGF-triggered actin polymerization. In this context, it is recognized that EGF-induced cell migration and EGFR activation in OSA cells are also associated to phosphorylation of ERK1/2, AKT, S6, and GSK3β, confirming activation of MAPK and PI3K/Akt downstream signaling pathways^30,31^. According to these data, PPN could be capable of blocking key protumoral signaling pathways involved in OSA growth and motility. However, the specific molecular mechanisms underlying these effects remain to be determined and further validated.

In our study, PPN anti-OSA activity was related to direct cytostatic effects and cell cycle arrest, decreasing survival and mitosis in ADRB2 expressing tumor tissue. However, SNS activation and β-adrenergic signaling can regulate a wide range of cancer-associated molecular pathways via both direct effects on ADRB expressing transformed cells and regulation of other ADRB-bearing cells populating the complex tumor stroma, such as adipocytes, fibroblasts, and vascular and immune cells^50,51^. In spite of using T cell-deficient nude mice for our in vivo studies, this animal model still has a robust innate immune response involving dendritic cells, tumor-associated macrophages and natural killer cells, which are known to play a key role against OSA^52^. Taking these concepts into account, other unexplored microenvironmental mechanisms associated with pleiotropic β-blockade, such as increased immune function, reversal of adipose tissue-driven inflammation or cancer-associated fibroblast inactivation, could be playing a part in the overall PPN activity and should not be discarded^53,54^. It is important to note that in the current study, concentrations in the range of 10–50 µM PPN were needed in order to significantly impair OSA aggressiveness in vitro. As mentioned, these concentrations are not achievable in vivo with standard dosage (plasma concentrations in PPN-treated patients can reach values of up to 500 ng/mL, or ≅ 2 µM)^46,55^. This suggests that the in vivo anti-tumor efficacy of PPN may not be the result of isolated direct cytostatic activity against malignant tissue, but rather through the sum of additional stroma-related mechanisms, such as angiogenesis inhibition. In accordance with an overwhelming body of evidence describing PPN as a highly efficient angiostatic agent^8,56^, we demonstrated that PPN, administered following a clinically-relevant dosage scheme of 10 mg/kg/day i.p., reduced early angiogenic response in MG-63 implants.

Enhancement of antineoplastic activity as a result of combining PPN with cytotoxic agents was previously reported for other tumor types, in which addition of repurposed drug to chemotherapy increased antiangiogenic and antiproliferative effects of monotherapies, as well as decreased distant metastases in patients^57–59^. Moreover, it was recently reported that β-blockade increases sensitivity to radiotherapy in different bone sarcoma models, especially in canine OSA^17^. In our in vivo protocol, sustained treatment with PPN using validated human equivalent doses (10 mg/kg/day i.p.), in addition to low-dose metronomic CDDP (2 mg/kg/day i.p.), significantly reduced the progression rates of OSA tumors, enhancing therapeutic benefits without increasing toxicity. Enhanced antineoplastic effectiveness of PPN and CDDP combination therapy could be associated with multiple mechanisms. First, it is known that low doses of CDDP induce cell cycle arrest in OSA, especially in highly proliferating MG-63 cells^60,61^, and that sensitivity of MG-63 tumors to CDDP therapy is highly dependent on cyclin D1 signaling^62^. Secondly, low-dose metronomic scheduling of CDDP is known to block tumor-driven angiogenesis triggering angiostatic mechanisms via modulation of TIMP-1 and different MMPs^63–65^. Considering the effects of PPN on angiogenesis and cell cycle progression, combination of this β-blocker with CDDP could favor a cooperative suppression of mitogenic potential of OSA cells as well as an inhibition of tumor-associated vasculature, resulting in impaired xenograft growth, reduced mitosis and enhanced tumor necrosis. Other potential mechanisms, such as modulation of P-gp function, could be playing a role in the observed cooperative benefits and should be considered in the future^40^. These findings bear high translational relevance given that necrosis rate in response to therapy and mitotic index determination are two histopathological parameters routinely used in the clinical management of OSA, linked to disease aggressiveness and prognosis^36,66^.

Our study has several limitations worth noting. It is widely recognized that in vivo models should recapitulate the tumor stroma as closer to the clinical setting as possible^67^. Given that PPN targets both tumor and its microenvironment, the influence of OSA tumor stroma should not be neglected, and the use of orthotopic paratibial or intraosseous tumor models should be considered for future studies. In addition, it is widely accepted that OSA is characterized by a complex genotype and high inter- and intra-tumor heterogeneity^68^. As a consequence, with the aim of reproducing this complex clinical heterogeneity, the use of OSA patient-derived xenografts should be addressed as a priority. Finally, although we show that PPN treatment affects different metastases-associated cellular traits in vitro, such as 2D/3D colony growth or tumor cell chemotaxis, effects of PPN administration on in vivo metastatic spread and outgrowth, especially to lungs, should be evaluated in order to fully characterize its therapeutic potential in OSA.

OSA is fatal for about one-third of the patients worldwide. However, prognosis is significantly poorer for LMICs^69^. Higher mortality rates are mainly due to economic inequalities and inefficient health-care systems, resulting in diagnosis at advanced stages and poor access to treatment resources, including surgery, radiotherapy and high-cost novel cancer drugs^70^. As previously introduced, *metronomics* combines drug repurposing with metronomic chemotherapy and could act as a substitute for standard treatments when unavailable or undoable^69^. PPN is a low-cost widely-available FDA-approved drug and its repositioned use in oncology is gaining strength as high quality preclinical and clinical evidence accumulates. On the other hand, in the past years metronomic chemotherapy has emerged as a promising multi-targeted therapeutic approach, showing clinical benefits in aggressive pediatric tumors with low associated-toxicities and used in outpatient settings^71–75^. Therefore, the combination of metronomic chemotherapy with repurposed PPN seems to deserve further research in OSA especially in developing countries. This approach could not only improve the effectiveness of chemotherapy but also contribute to reducing severe CDDP-related adverse effects.

## Conclusions

**Figure 8:**
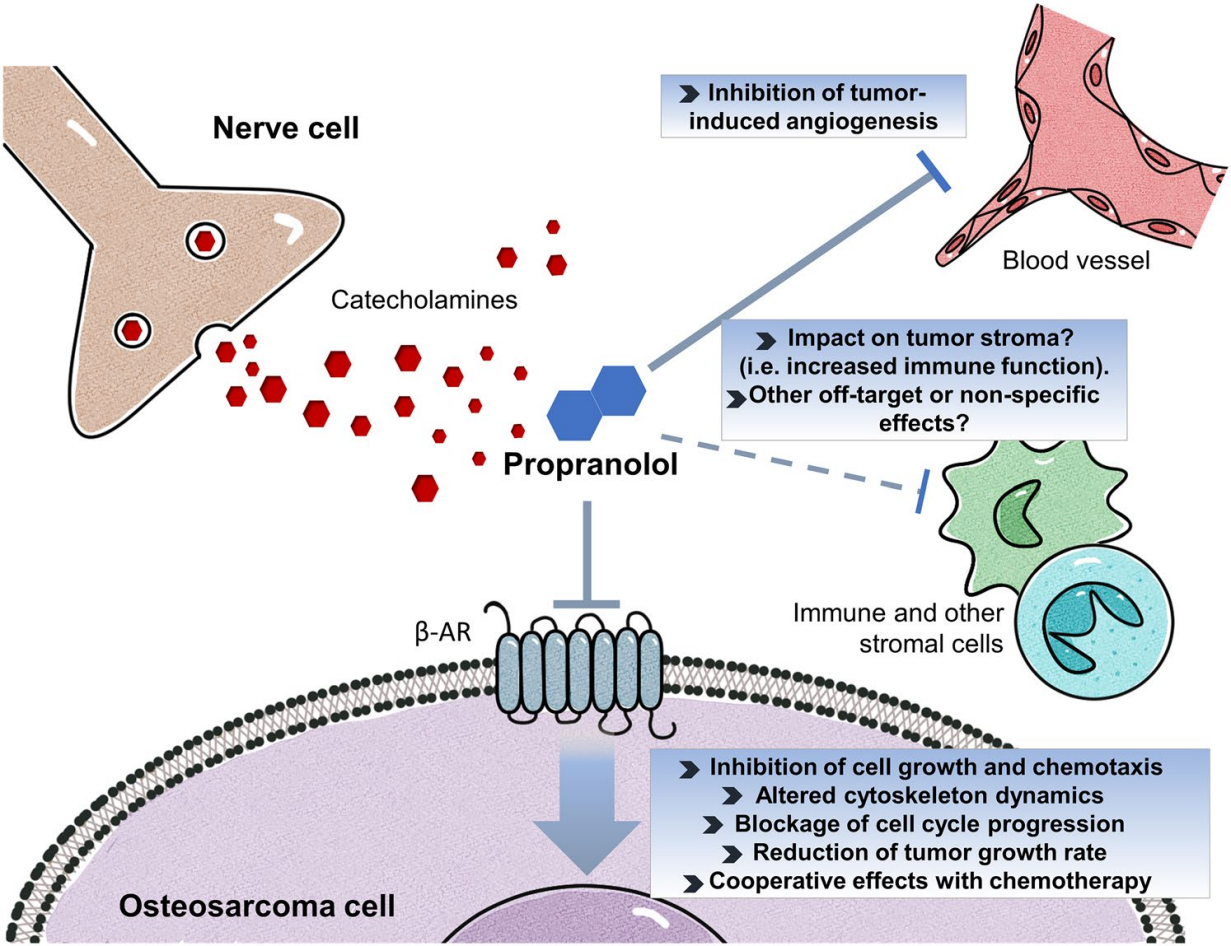
Potential antitumor activity and therapeutic benefits of repurposed β-blocker propranolol in osteosarcoma. β-AR signaling is involved in cancer progression and is upregulated in osteosarcoma (OSA). Its activation by catecholamines in tumor cells, as well as in other stromal players such as vascular and immune cells (i.e. neutrophils, macrophages, T cells, among others), promotes cell growth and angiogenesis, favors the formation of immune permissive niches and accelerates tumor progression. Besides antagonizing the growth-stimulating effects of catecholamines, treatment of ADRB2-expressing OSA cells with β-blocker propranolol (PPN) seems to inhibit cellular growth and chemotaxis by blocking cell cycle progression and altering actin cytoskeleton dynamics. PPN treatment could complement chemotherapy by inhibiting OSA-induced angiogenesis, enhancing tumor necrosis and thus delaying disease progression. With the aim of advancing PPN repurposing towards clinical practice further studies regarding its anti-OSA mechanisms of action are warranted, including its potential impact on tumor immune microenvironment and metastatic spread.

Survival rates of OSA patients have remained practically unaltered in the last 30 years, highlighting the need of intensifying research efforts and drug development programs to tackle this disease. β-adrenergic antagonization seems an achievable and interesting therapeutic approach to inhibit OSA progression (Fig. 8). Integration of novel therapeutic approaches such as the use of repurposed PPN into current OSA management is both promising and challenging. We believe that this study could help lay the groundwork for translating the use of PPN as a novel adjuvant therapy in OSA.

## Methods

### Drugs and compounds

PPN hydrochloride lyophilized powder (Sigma-Aldrich, Missouri, USA) was first solubilized using citrate buffer (pH 3) and further diluted with phosphate buffer saline (PBS) to reach working concentrations. CDDP was purchased from Abbot laboratories (Illinois, USA). Catecholamines EPI and NOR were purchased from BIOL laboratories (Buenos Aires, Argentina).

### Tumor cell lines

Human MG-63 (ATCC CRL-1427) and U-2OS (ATCC HTB-96) OSA cell lines were grown in Dulbecco's modified Eagle's medium (DMEM) (Gibco, Massachusetts, USA) plus 10% fetal bovine serum (FBS) (Natocor, Córdoba, Argentina), 2 mM glutamine and 80 µg/ml gentamicin in monolayer culture, at 37˚C in a humidified atmosphere of 5% CO_2_. All cells were harvested using a trypsin/EDTA solution (Gibco, Massachusetts, USA) and routinely tested for mycoplasma. Human glioma U-87MG (ATCC HTB-14) and LN-229 cells (ATCC CRL-2611), and human prostate PC-3 cells (ATCC CRL-1435) were included as control cellular models in different experimental procedures.

### OSA cell growth

Crystal violet assay was used for assessing OSA cellular growth. Briefly, MG-63, U-2OS or U-87MG cells were plated in 96-well flat bottom plates at a density of 2.5 × 10^3^ or 5 × 10^3^, respectively, per 100 µL in complete DMEM, allowed to attach overnight, and then treated with PPN, catecholamines, dobutamine, CDDP or vehicle for 72 h. Then cells were fixed for 10 min with methanol and then stained with 0.5% crystal violet solution for another 10 min. Following a washing step with deionized water, fixed cultures were dried overnight. Quantification was performed by direct counting or absorbance measurement (λ = 595 nm) using a multiplate reader after adding an ethanol/acetic acid 3:1 solution. Values are presented as a percentage of control. For the calculation of the half maximal inhibitory concentration (IC_50_), linear and nonlinear regressions were applied, depending on optimal curve-fitting. For synergy assessment in drug combination studies, the nature of the interactions between studied compounds was analyzed using the combination index (CI) method calculated in the Compusyn software v1.0 (Combosyn Inc., New Jersey, USA, www.combosyn.com). For the quantitative evaluation of this interaction we used full-range dose–response curves for each evaluated drug. Synergy was determined when CI was < 1.

### Transient transfection with ADRB2-targeting siRNA

For transfection, 2.5 × 10^3^ MG-63 cells were seeded per well in 96 well-plates in complete medium. After 24 h, knock-down was conducted using double stranded small interfering RNA (siRNA) targeting ADRB2 as previously described^76,77^. For increased knock-down efficiency, a 10 nM mixture of three different sequences of ADRB2-targeting siRNA was used. Non-targeting scrambled siRNA at 10 nM was used as a control. ADRB2 siRNAs and non-targeting controls were kindly provided by Dr. Ariana Bruzzone (Biochemical Research Institute, CONICET-National University of the South, Bahía Blanca, Argentina). Transfection was performed using Lipofectamine 2000 reagent (Invitrogen, Thermo Fisher, Massachusetts, USA) according to the manufacturer's instructions. After 6 h, the transfection medium was removed and replaced by fresh complete growth medium. In order to evaluate the impact of ADRB2 knock-down on drug response, transfected cells with ADRB2 or scrambled siRNA were incubated with PPN (50 µM) or saline vehicle for 72 h. OSA tumor cell growth was assessed as previously described. Efficiency of knockdown in tumor cells by ADRB2-targeting siRNAs was assessed and confirmed after 72 h of transfection^76^.

#### Cytotoxicity assay

For cytotoxicity assessment of short-term exposure to PPN, semiconfluent monolayers of MG-63 cells were treated with saline vehicle (control) or PPN (10 or 50 µM) for 24 h. After treatment, cells were harvested and the viable cells were counted in a Neubauer improved cell counting chamber using trypan blue dye under a light microscope. Percentage of cell viability and total number of viable cells were calculated.

#### Quantitative real time polymerase chain reaction

Total RNA from 1 × 10^6^ MG-63 cells was purified with Trizol. RNA was reverse transcribed with MMLV Transcripta (Productos Bio-Lógicos, Buenos Aires, Argentina) according to the manufacturer's protocol. The following specific forward and reverse primers were used: for β2-adrenergic receptor (ADRB2), 5’-TTTTGGCAACTTCTGGTGCG-3´ and 5´-GATCACGCACAGGGTCTCAA-3´; for β-actin (ACTB), 5´-GGACTTCGAGCAAGAGATGG-3´ and 5´-AGGAAGGAAGGCTGGAAGAG-3´; for cyclin-D1 (CCND1), 5´-TGGTGAACAAGCTCAAGTGG-3´ and 5´-CTGGCATTTTGGAGAGGAAG-3´. The expression of CCND1 was measured in 48 h serum starved MG-63 cells treated with PPN 50 µM for 24 h, as stated in “cell cycle distribution” methodology. Quantitative real time polymerase chain reaction was performed using iTaq Universal SYBR Green Supermix (Bio-Rad Laboratories Inc., California, USA) and CFX96 Touch Real-Time PCR Detection System (Bio-Rad). The following thermal cycling conditions were used: 95 °C for 10 min, 40 cycles of 95 °C for 15 s followed by 60 °C for 60 s. Each sample was analyzed in triplicate and mean cycle threshold values (Ct) were used for further analysis. Ct values were normalized for ACTB expression levels and normalized to control samples. Relative quantification values were calculated as 2.

### Western Blot

MG-63 cells were plated on 6-well culture dishes in growth medium. After 24 h starvation, cells were treated for 5, 10, 30, 60 or 120 min with 10 nM of catecholamines EPI and NOR. After treatment, cells were washed twice with PBS and protein extracts were prepared by homogenizing an equal number of cells in ice-cold RIPA buffer containing protease and phosphatase inhibitors (P8340 and P0044, Sigma-Aldrich, Missouri, USA). Protein concentrations were determined by Bradford assay. Samples were separated by 10% SDS-PAGE and electro-transferred onto polyvinylidene difluoride membranes. After blockade with 5% non-fat milk in TBST buffer, membranes were incubated with primary antibodies at 4 °C overnight, followed by anti-rabbit IgG 1:1000 horseradish peroxidase (HRP)-conjugated secondary antibody (7074, Cell Signaling Technology Inc., Massachusetts, USA) for 1 h. Immunoreactive signals of target proteins were detected with ECL™ Prime Western Blotting System (Cytiva RPN2232, Sigma-Aldrich) and images were taken using the ChemiDocChemiluminescence Western blot Scanner (Bio-Rad Laboratories Inc., California, USA) and analyzed with Image Lab software (Bio-Rad Laboratories Inc.). The following primary antibodies were used: p44/42 MAPK (ERK1/2) (137F5) Rabbit mAb (4695S), Phospho-p44/42 MAPK (ERK1/2) Rabbit mAb (4370S), α-Tubulin (2144) (Cell Signaling Technology Inc.).

#### Cell cycle distribution

After 48 h of serum starvation, MG-63 and U-2OS cells were treated for 24 h with PPN (50 µM) in complete DMEM and collected by trypsinization. Cells were then fixed in 70% chilled methanol over-night, treated with 1 μg/ml RNase A (Sigma-Aldrich, Missouri, USA) and stained with 100 μg/ml propidium iodide (Thermo Fisher, Massachusetts, USA). Cell cycle phase distribution of nuclear DNA was carried out in a FACSCalibur cytometer (Becton Dickinson, New Jersey, USA) using FlowJo v7.6.2 software (BD Life Sciences, New Jersey, USA, www.flowjo.com). Cells with PI staining intensity lower than the G_0_/G_1_ peak were considered hypodiploid. Increase in the sub-G_0_/G_1_ cell population was used to estimate apoptosis due to their hypodiploid DNA content.

#### Apoptosis

1 ×  10^4^ MG-63 or 1.5 × 10^4^ U-2OS cells were plated on glass coverslips resting on 24-well flat bottom plates and allowed to attach overnight. After 24 h starvation MG-63 cells were treated for 48 h with PPN (50 µM) in complete DMEM. After incubation, cells were fixed in 4% paraformaldehyde in PBS. DNA strand breaks were labeled with digoxigenin-dUTP using terminal deoxynucleotidyl transferase nick end labeling (DeadEnd TUNEL System, Promega, Wisconsin, USA), according to the manufacturer’s protocol. Slides were mounted using DAPI-containing Vectashield mounting medium (Vector Laboratories Inc., California, USA) for DNA staining and visualized using fluorescence microscopy in a Cytation Gen5 Cell Imaging Multi-Mode Reader (BioTek, Vermont, USA). 10 pictures per experimental group were taken for quantification. The number of apoptotic cells evaluated by TUNEL was expressed as [number of TUNEL-positive cells/total cells] × 100 in each specific condition (± 95% confidence limits), and analyzed by the χ^2^ test.

#### In vitro* mitosis*

PFA-fixed and DAPI-stained OSA cell cultures, MG-63 and U-2OS, were analyzed for mitotic index calculation after a 48 h exposure to PPN (50 µM). Stained slides were visualized and photographed using a Cytation Gen5 Cell Imaging Multi-Mode Reader (BioTek, Vermont, USA). Mitotic figures were counted using a high power field (HPF) of 400-fold magnification (×400) and the percentage of mitotic cells was obtained after quantification of total cell number. Stages of mitosis that were included were representative of prophase through telophase. Ten pictures per group were used for quantification.

#### Clonogenic growth

Clonogenic assays were conducted in order to evaluate the effect of PPN on OSA cell 2D colony formation. 250 MG-63 cells/well or 500 U-2OS cells/well were plated on 96-well flat bottom plates and cultured for 7 days in complete DMEM which was replaced every 72 h with PPN or vehicle. At the end of the week, OSA colonies were fixed and stained as reported to facilitate quantification. For MG-63 cells, proximate and discrete cellular aggregates involving more than 20 cells were considered tumor cell colonies. Representative pictures of each well were taken at ×40 magnification (Nikon Microscope TE-2000 and NIS-Elements 3.0 software, Tokyo, Japan) and colony quantification was conducted per well using the open-source software QuPath v0.3.0 (QuPath, Quantitave Pathology and Bioimage Analysis, University of Edinburgh, UK, qupath.github.io)^78^.

#### Cell proliferation

Additionally, the impact of PPN on the proliferation of low-density MG-63 cultures was measured using the metabolic MTS assay (Sigma Aldrich). MTS was added to each well and after a 4 h incubation, direct measurement of absorbance at 490 nm was conducted. The optical density of untreated control cells was taken as 100% viability.

#### OSA spheroid growth

The hanging drop method was used in order to generate multicellular OSA tumor spheroids. 20 µl drops containing a cellular suspension of 2.5 × 10^3^ MG-63 cells in complete DMEM were placed on the top cover of a 100 mm plate. After 72 h, cell aggregates were gently placed in 96-well plates coated with 50 µl of 1.5% agar. After one week, when spheroids acquired an adequate morphology and a minimum diameter of 200 µm, cells were exposed to 50 µM PPN during 7 days. Complete DMEM with or without PPN was replaced every 72 h. Pictures of tumor spheroids were taken using a high-resolution optic microscope (Leica Microsystems, Wetzlar, Germany) at three different timepoints. At the end of the experiment, ReViSP v2.2 (The Math Works Inc., Massachusetts, USA) and AnaSP v1.4 softwares (The Math Works Inc., Massachusetts, USA) were used to evaluate volume and confirm sphericity of the spheroids^79,80^.

#### Cell chemotaxis

After overnight starvation, 2.5 × 10^4^ MG-63 or U-2OS cells were seeded into the 8 µm-pore inserts (JetBiofil, Guangzhou, China) in serum-free DMEM. Lower chambers were filled with 10% FBS-containing DMEM as chemoattractant. After 24 h-incubation with 50 µM PPN, cells present in the upper surface of the membranes were removed with cotton swabs and cells that migrated through the insert and attached to its lower surface were fixed, stained and washed. Migrating cells in five randomly selected ×400-HPF were counted and normalized to control.

#### Tube formation assay

A Matrigel-based morphogenesis assay was used to assess capillary-like tube formation, also known as vasculogenic activity, by OSA cells^81^. MG.63 cells were chosen given their capacity for rapid tube formation^82^. Briefly, wells of 96-well flat bottom plates were coated with 80 μL Growth Factor Reduced Matrigel (Corning, New Jersey, USA) at 37 °C for 15 min according to the thin gel method (manufacturer’s protocol). Cells were seeded at a density of 1.5 × 10^4^/well in 50 µl and then another 50 µl were added with PPN in order to achieve a final concentration of 50 µM. Well-defined patterned networks were observed after overnight incubation. After 16 h closed tubular structures in four randomly selected ×100-HPF per assay were counted and normalized to control. Glioma LN-229 and U-87MG cells were used as negative and positive vasculogenic mimicry control cells, respectively (Data not shown)^83^.

#### Actin fluorescent staining

MG-63 cells grown in glass coverslips were incubated overnight for 20 h in serum free DMEM, treated for 1 h with PPN (10 and 50 µM) and then stimulated with EGF (100 ng/ml) (Life Technologies, Thermo Fisher, Massachusetts, USA) for 20 min. Cells were fixed in 3.7% formaldehyde in PBS and stained with phalloidin conjugated with AlexaFluor555 in order to analyze actin cytoskeleton (Life Technologies) following the instructions of the manufacturer. Cell nuclei were detected with DAPI. Phalloid-stained actin was assessed using a TCS SP8 confocal laser scanning microscope (CLSM, Leica Microsystems, Wetzlar, Germany) operated by the Leica Application Suite X program (LAS × V3.7.2, Leica Microsystems). Twelve-bit images with 4096 × 4096-pixel resolution were obtained by sequential scanning using a ×63 oil immersion objective. LAS × software was used for analysis of digitized CLSM images in order to calculate the percentage of actin stress fiber rich-cells or cells with rounded morphology, in each ×630 HPF. For statistical comparison between experimental groups, contingency analysis and Fisher's exact test were conducted. In addition, two independent trained operators used a semi-automated hand-drawn polygon contouring system to delineate specific ROIs for cellular area quantification. Single cell values were averaged for a minimum of 30 cells per experimental condition (6–7 cells per high power field).

#### Animals

Outbred athymic female N:NIH(S)-nu mice aged 8 weeks with a weight of approximately 23 g, were purchased from the School of Veterinary Sciences Animal Facility at National University of La Plata (Buenos Aires, Argentina) and, after randomization, housed at 5 mice per cage in our animal facility at the National University of Quilmes. Food and water were provided ad libitum and the general health status of the animals was monitored daily.

#### Modified Matrigel plug assay

To evaluate the effects on OSA-induced angiogenesis, a modified Matrigel plug assay was conducted. A mixture containing 400 µl of Matrigel, heparin (50 U/ml) and 4 × 10^6^ MG-63 cells in 100 µl serum-free DMEM medium was injected subcutaneously into athymic mice. Treatment consisted of five daily consecutive doses of PPN 10 mg/kg i.p. This clinically relevant dosing scheme was adopted from previously reported preclinical studies^59^ and is in range with clinical references (including pediatric population)^84^ after “human to mice” dose conversion. This calculation is performed by dose extrapolation using the ‘dose by factor’ method based on allometric scaling, following the United States Food and Drug Administration guidelines^85^. Animals were sacrificed 7 days after cell injection. Plugs were recovered and the extent of vascularization was assessed by the amount of hemoglobin detected in the implants using the Drabkin method (Sigma-Aldrich, St Louis, MO, USA)^86,87^. The mean optical density of plugs from the control group was taken as 100% (relative hemoglobin content). Representative plugs were photographed before processing for hemoglobin quantification.

#### OSA xenograft progression

Human OSA tumors were heterotopically generated after subcutaneous injection of a 150 µl suspension containing 5 × 10^6^ MG-63 cells in DMEM and Matrigel (Corning, New Jersey, USA) in a 2:1 volume ratio MG-63 cells in athymic mice^88^. Tumors were measured periodically with a caliper and tumor volume was calculated by the formula: 0.52 × width^2^ × length. During the protocol animal weights and tumor growth rates (TGR) were also assessed. TGR represents the slopes of the linear regressions of the tumor volumes over time. Treatment schedules started 3 days after cell inoculation, when tumors were detected by palpation. When first signs of skin infiltration appeared on primary lesions and larger tumors reached a volume of 300 mm^3^, animals were photographed and protocol was ended. Animals were euthanized by cervical dislocation and tumors were removed, weighted, fixed with formalin and processed for hematoxylin and eosin (H&E) staining. In vivo combination studies were performed by administering PPN (10 mg/kg i.p.) in a 5-day-on/2-day-off schedule, alone or in combination with CDDP (2 mg/kg i.p.) during 4 weeks. Once again, PPN^59^ and CDDP^89^ dosage was defined according to previously reported preclinical studies. Chemotherapy was used following a metronomic scheduling consisting in sustained administration of low-dose CDDP three times per week during 4 weeks, without drug-free intervals until the end of the protocol^35,64^. Tumor growth rates and volume, as well as total animal weight were recorded or calculated throughout the protocol. Histopathological assessment of OSA tumors involved mitotic index quantification in viable sections of H&E-stained tumor slides and determination of adjusted tumor necrotic rate after treatment^32^. Mitotic bodies in H&E-stained slides were counted in 16 randomly-selected HPF (×400). Only viable sections of tumor tissue were analyzed for mitotic index calculation. Histological analysis was performed and confirmed by two blinded researchers. For tumor necrosis assessment, color brightfield images of entire H&E-stained tumor sections were acquired at ×2.5 magnification using a Cytation Gen5 Reader (BioTek, Vermont, USA). Images were collected using a 4 × 5 grid and the stitching was performed with the “Image Montage” function setting a tile overlapping of 10%. Necrotic area in tumor tissue sections was measured using ImageJ 1.5j8 Software (NIH, Maryland, USA, imagej.nih.gov). Tumor necrosis was identified as tissue areas with a marked increase of eosinophilia and quantification of both necrotic area (NA) and viable area (VA) was performed using the “Color Threshold’’ tool. Tumor necrotic rate (TNR) was then calculated as: “TNR = (NA * 100) / (VA + NA)” in 4 sections per experimental group. Adjustment of % of necrotic areas to changes in tumor size and determination of adjusted tumor necrotic rate (ATNR) was performed using the following equation: “ATNR = 100–(100–TNR) x RTGR”, where RTGR stands for group-specific relative tumor growth rates. RTGR was obtained after transforming TGR values, taking the TGR of the control group (6.8 mm3/day) as “1”. As a result, RTGR of 0.75, 0.94, and 0.34 were respectively used for necrosis adjustments in PPN, CDDP and PPN + CDDP-treated tumor slides.

#### Statistics

Statistical analysis was performed using the GraphPad Prism v6.0.0 (GraphPad Software Inc., San Diego, CA, USA, www.graphpad.com) or Compusyn software v1.0 (Combosyn Inc., New Jersey, USA, www.combosyn.com). To compare differences between two experimental groups Mann Whitney or t tests were used for non-parametric or normal distribution of data, respectively. In case of more than two experimental groups, ANOVA analysis with Tukey's multiple comparisons post-test was used when normal distribution of data was determined. Kruskal–Wallis analysis with Dunn's multiple comparisons post-test was used in case of non-parametric distribution of data. Differences were considered statistically significant at a level of *p* < 0.05. Data corresponds to at least 2 or 3 independent experiments unless stated otherwise. Data were presented as mean ± standard deviation (SD) or standard error of mean (SEM).

### Ethical approval

All animal protocols have been carried out in accordance with the Guide for the Care and Use of Laboratory Animals as adopted by the U.S. National Institutes of Health (NIH Publications No. 8023, revised 1978) and were approved by our institutional Animal Care Committee UNQ-CICUAL (Resolution CD CyT No. 075/14); Protocol codes: 007-15 and 011–15. Results from animal studies were reported following ARRIVE guidelines.

## Supplementary Information

Supplementary Information.

## Data Availability

The datasets used and/or analyzed during the current study are available from the corresponding author on reasonable request.
