# Supplementary material for: Propranolol blocks osteosarcoma cell cycle progression, inhibits angiogenesis and slows xenograft growth in combination with cisplatin-based chemotherapy
Source: Sci Rep. 2022 Sep 8;12:15058. doi: 10.1038/s41598-022-18324-3 (PMC9458647; doi:10.1038/s41598-022-18324-3)
Supplement: Supplementary file 1 — Supplementary Information. [file 41598_2022_18324_MOESM1_ESM.docx]

**Supplementary figure 1**


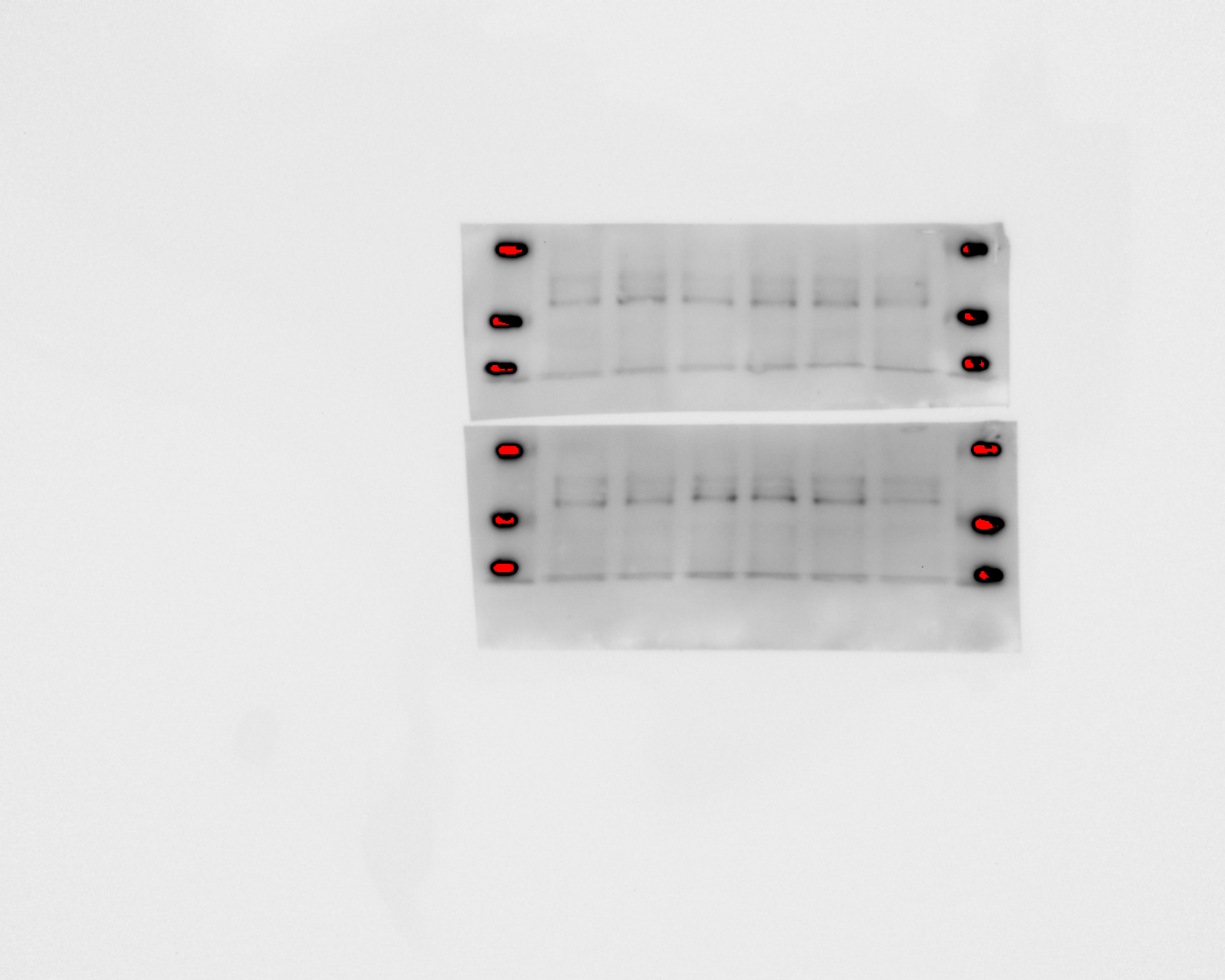


52 Kd

38 Kd

32 Kd

p-ERK

Control

5 ´

30´

10´

60´

120´

EPI+NOR 10 nM


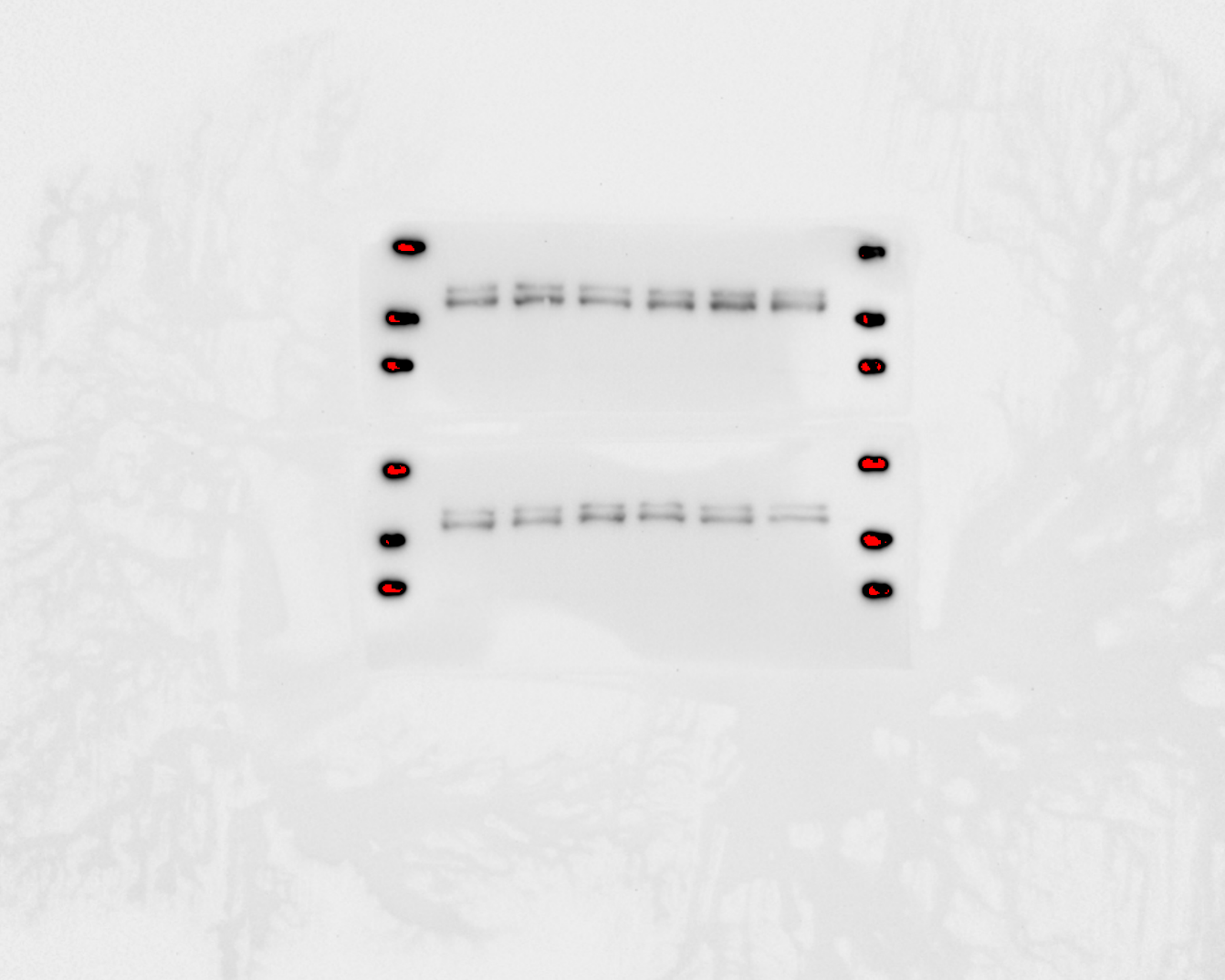


52 Kd

38 Kd

32 Kd


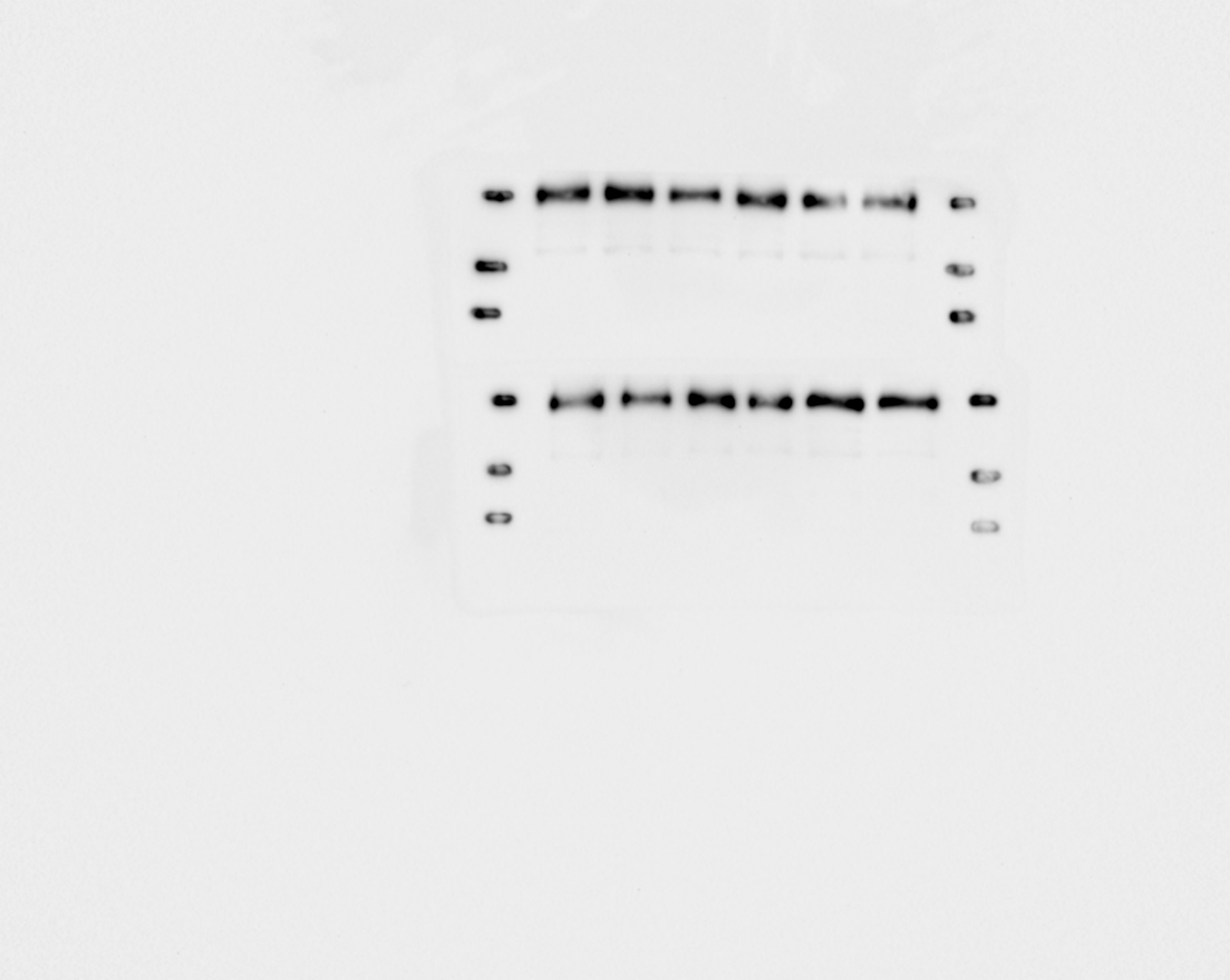


52 Kd

38 Kd

32 Kd

ERK

a-Tubulin

**Figure S1.** Western blot analysis for p-ERK, ERK and a-Tubulin protein expression in vehicle (control) or catecholamine treated MG-63 osteosarcoma cells. Original uncropped blots are depicted.

**Supplementary figure 2**

**Figure S2. Lack of growth-stimulating activity by dobutamine on osteosarcoma cells.** Tumor cell growth was evaluated on log-phase growing osteosarcoma (OSA) MG-63 cells and measured by crystal violet staining after a 72 h exposure to ADRB1 selective agonist dobutamine (1-1000 nM). ANOVA followed by Tukey’s test.

**Supplementary figure 3**

**Figure S3. Propranolol inhibits low density proliferation of osteosarcoma cells.** Low density MG-63 cell cultures were treated with PPN (1-50 µM) for 7 days (treatment was replaced every 72 h). In the end, proliferation of cells was measured using the metabolic MTS assay and direct measurement of absorbance at 490 nm was conducted. The optical density of untreated control cells was taken as 100% viability. *p<0.05, ***p< 0.001 and ****p< 0.0001. ANOVA followed by Tukey’s test.

**Supplementary figure 4**

**
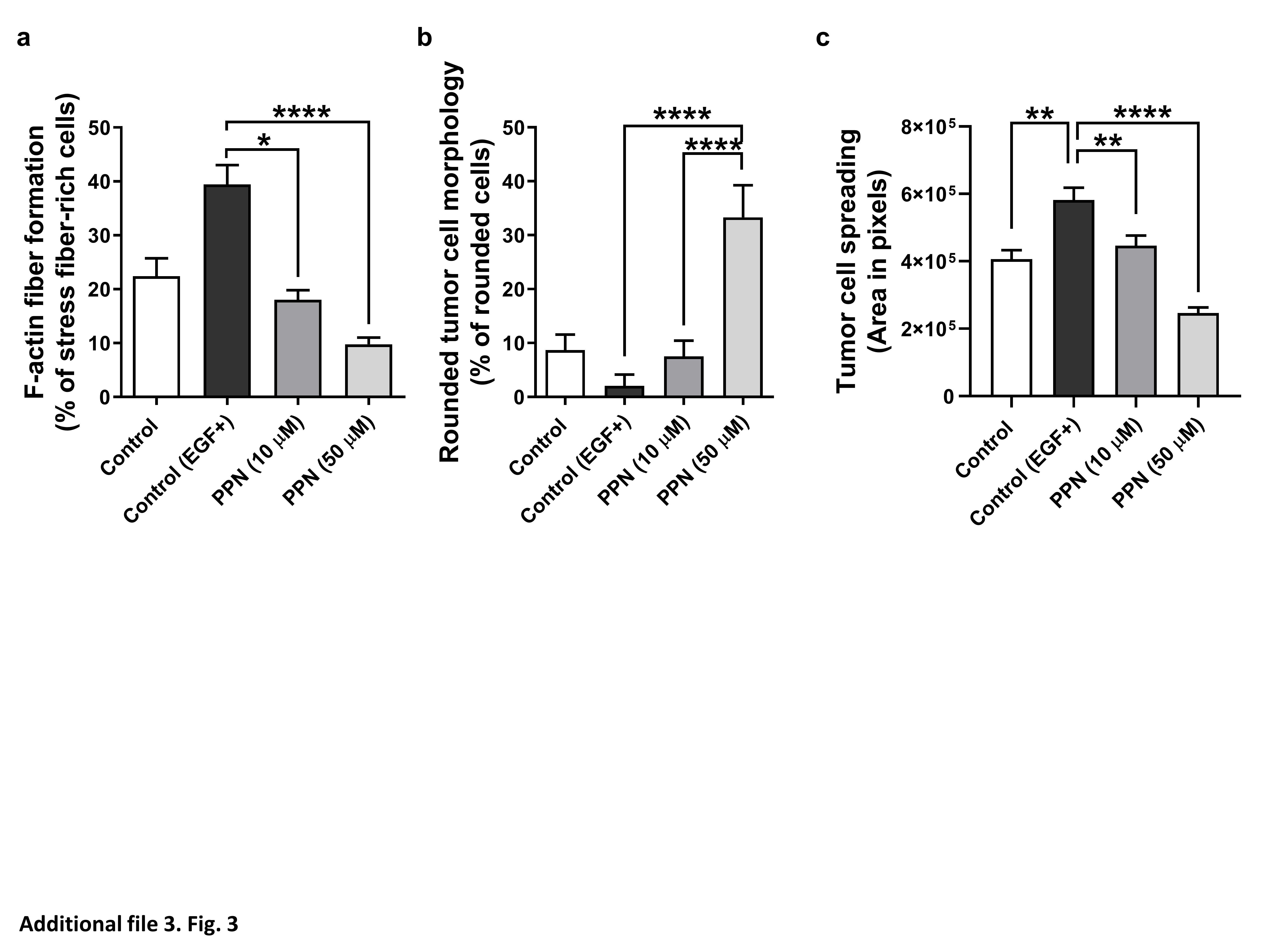
**

**Figure S4.** Propranolol treatment alters actin cytoskeleton dynamics and morphology in osteosarcoma cells. Actin cytoskeleton reorganization and stress fiber formation in MG-63 cells was induced by stimulation with epidermal growth factor (EGF) and evaluated using confocal laser scanning microscopy. (a) Percentage of stress fiber-rich cells in unstimulated (Control), or EGF-stimulated groups treated with vehicle (Control (EGF+)) or PPN (10 and 50 µM). (b) Percentage of tumor cells with rounded morphology in different experimental groups. (c) Quantitation of cellular area. *p<0.05, ***p< 0.001 and ****p< 0.0001. Contingency analysis and Fisher's exact test for (a) and (b), and ANOVA followed by Tukey’s test for (c).
